# Implantation awakens peri-implant osteogenic potential via Snx5-EGFR axis-mediated mechanical transduction

**DOI:** 10.1038/s41368-025-00423-2

**Published:** 2026-02-20

**Authors:** Xue Jiang, Yuteng Weng, Yanhuizhi Feng, Jie Huang, Haicheng Wang, Zuolin Wang

**Affiliations:** 1 Shanghai Engineering Research Center of Tooth Restoration and Regeneration & Tongji Research Institute of Stomatology, Shanghai, China; 2 Department of Oral and Maxillofacial Surgery, Department of Oral Implantology, Shanghai Tongji Stomatological Hospital and Dental School, Tongji University, Shanghai, China

**Keywords:** Experimental models of disease, Growth factor signalling

## Abstract

Alveolar bone resorption during the socket healing process compromises subsequent restoration outcomes. Recent clinical evidence suggests that dental implant placement can effectively prevent such bone loss, yet the mechanisms remain elusive. In this study, combined multi-dataset screening pinpointed sorting nexin 5 (Snx5) as a potential regulator of mechanotransduction, whose expression was downregulated in early peri-implant bone remodeling zones following implant placement. Functional studies showed that loss of Snx5 abolished the additional osteogenic enhancement normally induced by mechanical stimulation. In vivo, Snx5 deficiency disrupted the mechanosensitive activation of LepR^+^ MSCs and compromised implant-induced osteogenesis. Mechanistically, Snx5 facilitates the recycling of phosphorylated EGFR (p-EGFR) back to the plasma membrane to sustain EGFR signaling. Loss of Snx5 redirects EGFR trafficking toward late endosomes and lysosomal degradation, thereby weakening its signaling. These findings uncover a previously unrecognized role for Snx5 in mediating the osteogenic fate of peri-implant BMSCs in response to mechanical cues, expanding the functional repertoire of the Snx family. Collectively, these findings highlight Snx5 as a novel regulator of mechanosensitive bone remodeling and suggest that its downregulation may contribute to peri-implant bone adaptation. This study provides new insights into how the mechanical microenvironment regulates bone repair and highlights Snx5 as a promising molecular target for modulating skeletal mechano-responsiveness in clinical bone regeneration.

## Introduction

In clinical implant dentistry, post-extraction alveolar bone resorption remains a major challenge impacting long-term implant success. Extensive studies have demonstrated that within 3–6 months of natural healing following tooth extraction, the alveolar ridge undergoes significant vertical and horizontal bone loss, thereby complicating subsequent implant placement and reducing its predictability.^1^ Recent clinical evidence suggests that implant placement itself can delay this resorptive process and help maintain ridge contour.^2–4^ Interestingly, this bone-preserving effect appears to occur independently of early functional loading or prosthetic restoration,^5,6^ implying that immediate implant insertion may exert direct biological effects on the surrounding bone tissue through local mechanical or cellular mechanisms. However, the underlying cellular and molecular basis remains poorly understood.

Accumulating evidence indicates that mechanical stimuli regulate stem cell fate decisions and are actively involved in bone remodeling and metabolism.^7–9^ During implant bed preparation, clinicians often create an osteotomy slightly narrower than the implant diameter to achieve optimal primary stability, thereby introducing localized mechanical cues to the peri-implant bone during the insertion phase. Finite element modeling further suggests that implant placement generates complex mechanical stimuli within the surrounding jawbone, particularly at the implant–bone interface.^10^ These non-physiological yet controlled mechanical cues may represent critical modulators of bone remodeling in the peri-implant environment.

Bone marrow mesenchymal stem cells (BMSCs) are key regulators of skeletal homeostasis and repair.^11,12^ Among them, a subpopulation marked by leptin receptor (LepR) expression has been identified as a principal source of osteoprogenitors in adult bone remodeling, characterized by multilineage differentiation capacity.^13,14^ Notably, LepR⁺ MSCs have been shown to sense mechanical stimuli within the jawbone and participate in local bone turnover.^13,15^ These findings suggest that LepR⁺ MSCs may serve as essential mechanoresponsive effectors in the unique biomechanical environment established following implant placement. However, how these cells behave and respond molecularly under real-world implant conditions remains largely unknown.

In this study, we identified sorting nexin 5 (Snx5) as a novel regulator of mechanotransduction in the context of dental implantation. Transcriptomic analyses of peri-implant LepR⁺ MSCs, peri-implant bone tissue, and BMSCs subjected to in vitro mechanical loading consistently revealed downregulation of Snx5, implicating its potential role in mechanotransduction. Functional assays showed that Snx5 deficiency eliminated the osteogenic enhancement normally induced by mechanical stimulation. Consistently, in vivo loss of Snx5 impaired implant-induced bone formation and reduced the mechanosensitive activation of LepR⁺ cells. Mechanistically, Snx5 facilitates EGFR recycling to the plasma membrane following endocytosis, thereby preventing its lysosomal degradation. Snx5 deficiency leads to sustained EGFR degradation, thereby affecting the expression of osteogenic proteins and the differentiation potential of BMSCs. These findings reveal a critical bridging role of Snx5 in linking mechanical cues to BMSCs–mediated osteogenesis.

Together, these findings, inspired by the clinical phenomenon that implants help preserve alveolar bone even without loading, uncover a previously unrecognized role of Snx5 in regulating osteogenesis of LepR⁺ MSCs under mechanical cues. This work expands our understanding of jawbone mechanobiology and provides a potential molecular target to improve bone preservation in implant therapy.

## Results

### Dental implant placement maintains alveolar bone height and promotes osteogenic differentiation, accompanied by downregulation of Snx5 expression in peri-implant LepR⁺ cells

**Fig. 1:**
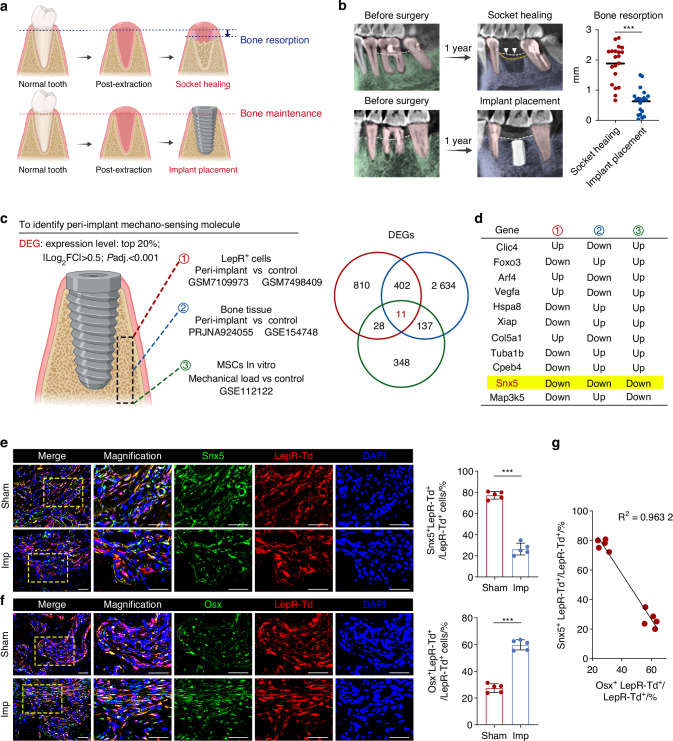
Dental implant placement maintains alveolar bone height and promotes osteogenesis, associated with downregulation of Snx5 in peri-implant regions. **a** Schematic diagram illustrating the effects of natural socket healing following tooth extraction and implant placement on alveolar bone height. **b** Representative radiographic images and quantitative analysis of bone resorption following natural healing or implant placement (*n* = 20). **c** DEGs were identified by integrating three transcriptomic datasets: (1) LepR⁺ cells from peri-implant and control regions (GSM7109973 and GSM7498409); (2) bulk RNA-seq data from peri-implant bone tissue (PRJNA924055 and GSE154748); and (3) MSCs under mechanical load in vitro (GSE112122). **d** Venn diagram showing 11 overlapping DEGs across the three datasets, with *Snx5* being the only gene consistently downregulated. **e** Immunofluorescence staining and quantification of Snx5 (green), LepR-tdTomato (red), and DAPI (blue) in peri-defect or peri-implant tissues from Sham and Imp groups. Overview and magnified views are shown on the left. Yellow dashed boxes indicate zoomed regions (*n* = 5). Scale bars: 100 μm. **f** Immunofluorescence staining and quantification of Osx (green), LepR-tdTomato (red), and DAPI (blue) in peri-defect or peri-implant tissues from Sham and Imp groups. Overview and magnified views are shown on the left. Yellow dashed boxes indicate zoomed regions (*n* = 5). Scale bars: 100 μm. **g** Correlation analysis between the number of Snx5⁺ LepR⁺ tdTomato⁺ cells and Osx⁺ LepR⁺ tdTomato⁺ cells (*n* = 10). RNA-seq RNA sequencing, DEGs differentially expressed genes, BMSCs bone marrow stromal cells, Imp implant, Data are presented as mean ± SD. ns not significant, *P < 0.05; **P < 0.01; ***P < 0.001; ****P < 0.000 1. Statistical analysis: two-tailed Student’s t-test for panels **b**, **e** and **f**; linear regression for **g** with the coefficient of determination (*R*²) shown

To evaluate the protective effect of dental implants on alveolar bone, we compared bone remodeling outcomes between extraction sockets with and without subsequent implant placement. Schematic illustrations and radiographic analyses demonstrated that alveolar ridge height progressively decreased during natural healing following tooth extraction, whereas dental implant placement effectively preserved bone mass, maintaining a favorable ridge height even one year postoperatively (Fig. 1a, b). In mouse models, immunofluorescence staining further revealed a significantly higher proportion of Runx2⁺ and Osx⁺ osteoprogenitor cells in peri-implant regions compared to the Sham group, indicating enhanced local osteogenic differentiation induced by implant placement (Fig. S1a, b).

To identify mechanosensitive molecules critically involved in implant-mediated bone preservation, we performed an integrative analysis of three independent transcriptomic datasets: (1) bulk RNA-seq data from FACS-sorted LepR⁺ cells from peri-implant and control bone tissue (GSM7109973 and GSM7498409); (2) bulk RNA-seq data from peri-implant and control bone tissue (PRJNA924055 and GSE154748); and (3) BMSCs subjected to in vitro compressive stress (GSE112122). Venn diagram analysis identified 11 overlapping differentially expressed genes (DEGs), among which *Snx5* was the only gene consistently downregulated across all three datasets (Fig. 1c, d). Immunofluorescence validation confirmed a significantly lower proportion of Snx5⁺ LepR⁺ tdTomato⁺ cells and a concomitant increase in Osx⁺ LepR⁺ tdTomato⁺ cells in the peri-implant regions compared to the Sham group (Fig. 1e, f). Furthermore, correlation analysis revealed a strong negative association between Snx5⁺ LepR⁺ tdTomato⁺ cells and Osx⁺ LepR⁺ tdTomato⁺ cells (*R*² = 0.9632), suggesting that downregulation of Snx5 may be closely linked to osteogenic differentiation of LepR⁺ progenitor cells (Fig. 1g).

### Snx5 deficiency reshapes the transcriptomic profile of BMSCs and modulates osteogenic responses under mechanical stimulation

**Fig. 2:**
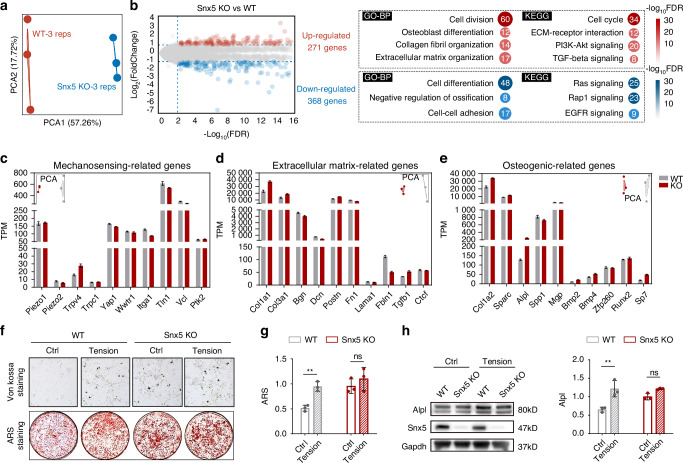
Snx5 deficiency reshapes the transcriptomic profile of BMSCs, enhances basal osteogenesis, but impairs osteogenic responses to mechanical stimulation. **a** PCA based on transcriptomic profiles of WT and *Snx5*-KO BMSCs (*n* = 3). **b** Volcano plot (left) showing upregulated genes (red) and downregulated genes (blue); GO-BP and KEGG signaling pathways enriched among differentially expressed genes (right). **c–e** TPM values for genes associated with mechanosensation (**c**), extracellular matrix components (**d**), and osteogenesis (**e**) between WT and *Snx5*-KO BMSCs. **f**, **g** Von kossa and ARS staining of WT and *Snx5*-KO BMSCs under static or mechanical tension conditions. **h** Western blot analysis of Snx5 and Alpl expression and quantification of Alpl levels in WT and *Snx5*-KO BMSCs under static or mechanical stimulation conditions (*n* = 3). PCA Principal component analysis, WT wild-type, KO knockout, GO-BP GO biological processes, KEGG kyoto encyclopedia of genes and genomes, TPM transcripts per million, ARS alizarin red staining. Data are presented as mean ± SD. ns not significant, *P < 0.05; **P < 0.01; ***P < 0.001; ****P < 0.000 1. Statistical analysis for **g** and **h** was performed using two-way ANOVA followed by Tukey’s multiple comparisons test

To investigate the impact of Snx5 deficiency on the transcriptomic landscape of BMSCs, we performed bulk RNA-seq on wild-type (WT) and *Snx5* knockout (*Snx5*-KO) BMSCs. Principal component analysis (PCA) revealed a clear separation between the two groups, indicating substantial transcriptomic differences (Fig. 2a). Differential expression analysis identified 271 upregulated and 368 downregulated genes in *Snx5*-KO BMSCs (Fig. 2b). Functional enrichment analysis demonstrated that upregulated genes were significantly enriched in osteogenic differentiation, extracellular matrix (ECM) remodeling, and pro-osteogenic GO and KEGG pathways such as PI3K–Akt and TGF–β signaling. Conversely, downregulated genes were associated with negative regulation of ossification and inhibitory pathways such as EGFR signaling (Fig. 2b).

While mechanosensitive genes such as *Piezo2* and *Yap1* showed a slight reduction in *Snx5*-KO cells, the differences were not statistically significant (Fig. 2c). In contrast, ECM-related genes (*Col1a1*, *Col3a1*) and osteogenesis-associated genes (*Alpl*, *Runx2*, *Sp7*) were significantly upregulated (Fig. 2d, e), indicating enhanced baseline osteogenic programming. Furthermore, Snx5 deficiency markedly upregulated proliferation-associated genes, including *Mki67*, *Top2a*, and *Pcna* (Fig. S2a), while the expression of stemness-related genes (*Sox2*, *Nanog*, *Prrx1*) remained largely unchanged (Fig. S2b). Consistent with the transcriptomic findings, alkaline phosphatase (ALP), Alizarin Red S (ARS), and von Kossa staining all demonstrated that Snx5 deficiency markedly enhanced the osteogenic capacity of BMSCs under basal conditions (Figs. 2f, g and S2c). However, upon application of mechanical tensile stress, WT cells exhibited a significant increase in osteogenic differentiation, whereas this response was impaired in *Snx5*-KO cells, as indicated by suppressed upregulation of Alpl protein levels (Fig. 2h). These results suggest that Snx5 deficiency compromises the enhancement of osteogenic responses induced by mechanical stimulation.

### Snx5 deficiency attenuates implant-induced osteogenesis and disrupts the mechanical responsiveness of LepR⁺ cells

**Fig. 3:**
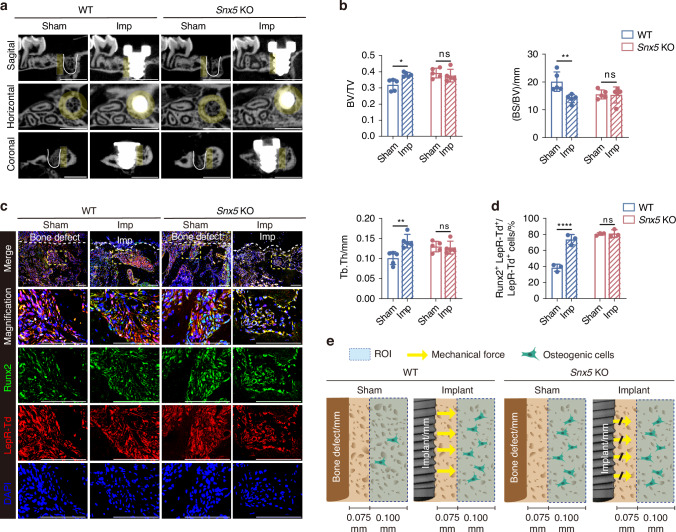
Snx5 deficiency impairs implant-induced osteogenesis and disrupts the mechanical responsiveness of LepR⁺ cells. **a** Representative micro-CT reconstructed images of bone architecture in WT and *Snx5*-KO mice under Sham or Implant conditions, showing sagittal, horizontal, and coronal views. Yellow areas indicate ROIs, defined as annular zones extending approximately 75 μm to 175 μm outward from the implant surface; white solid lines delineate the defect boundaries in the Sham group. Scale bar: 1 mm. **b** Quantitative analysis of BV/TV, BS/BV, and Tb.Th (*n* = 5). **c** Immunofluorescence staining and magnified views of peri-defect or peri-implant regions from Sham and Implant groups, showing Runx2 (green), LepR⁺ tdTomato⁺ (red), and DAPI (blue). Yellow dashed boxes indicate zoomed-in areas; white dashed lines outline the boundaries of the defect or implant sites (*n* = 3). Scale bars: 100 μm. **d** Quantification of Runx2⁺ LepR⁺ tdTomato⁺ cells as a percentage of total LepR⁺ tdTomato⁺ cells (*n* = 3). **e** Schematic illustration showing differential responses to implant-induced mechanical cues in WT and *Snx5*-KO mice: in WT mice (left), implant placement promotes osteogenic differentiation; in *Snx5*-KO mice (right), this process is impaired, resulting in reduced osteogenic response. WT wild-type, KO knockout, Imp implant, ROI regions of interest, BV/TV bone volume fraction, BS/BV bone surface-to-volume ratio, Tb.Th trabecular thickness. Data are presented as mean ± SD. ns not significant, *P < 0.05; **P < 0.01; ***P < 0.001; ****P < 0.000 1. Statistical analysis for **b** and **d** was performed using two-way ANOVA followed by Tukey’s multiple comparisons test

To investigate the role of Snx5 in implant-induced osteogenesis, we established a dental implant model in both WT and *Snx5*-KO mice and evaluated their bone regeneration capacity. No significant differences in body weight were observed between *Snx5*-KO and WT mice of either sex throughout the experimental period. In addition, *Snx5*-KO mice exhibited normal survival rates, comparable to those of WT controls (Fig. S4a, b). Micro-CT analysis revealed that, following implant placement, WT mice showed a marked increase in bone mass, whereas *Snx5*-KO mice did not exhibit further bone gain, suggesting that *Snx5* deficiency impairs implant-induced osteogenic responses (Fig. 3a, b).

Immunofluorescence staining was performed to further assess the osteogenic potential of LepR⁺ lineage cells in peri-implant regions. In WT mice, implant placement significantly reduced the proportion of Snx5⁺ LepR⁺ tdTomato⁺ cells, accompanied by an upregulation of Runx2⁺ LepR⁺ tdTomato⁺ osteogenic cells (Figs. 3c, d and S5a, b). In contrast, these changes were not observed in *Snx5*-KO mice, indicating that Snx5 plays a critical role in the mechanosensation and osteogenic conversion of LepR⁺ lineage cells.

A mechanistic illustration summarizes these findings: in WT mice, mechanical cues generated by the implant effectively drive the osteogenic differentiation of LepR⁺ cells, contributing to enhanced peri-implant bone formation. However, in the absence of Snx5, this mechanotransduction process is disrupted, leading to compromised bone regeneration in response to the implant (Fig. 3e).

### Snx5 regulates osteogenesis through EGFR signaling pathway, and EGF-mediated activation of EGFR reverses the pro-osteogenic effects of Snx5 deficiency

**Fig. 4:**
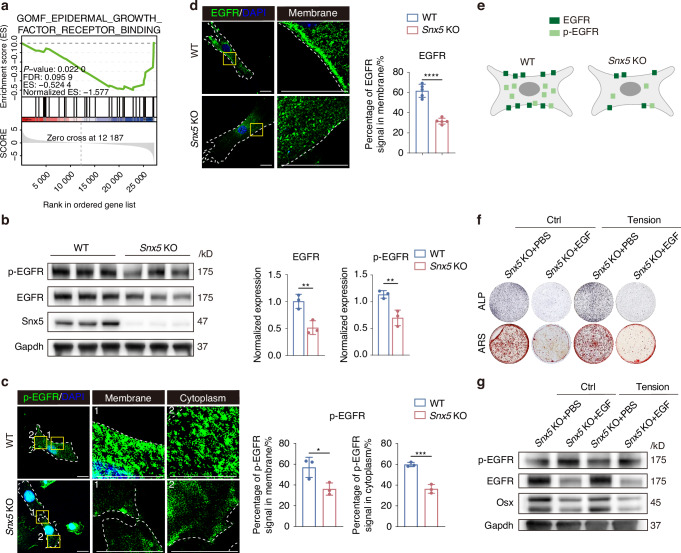
Snx5 regulates osteogenesis through modulation of EGFR signaling. **a** GSEA of gene sets related to EGFR signaling following *Snx5* knockout. The *p*-value, FDR, ES, and NES are shown. **b** Western blot analysis and quantification of total EGFR and p-EGFR protein levels following *Snx5* knockout (*n* = 3). **c** Immunofluorescence staining and magnified views of p-EGFR in WT and *Snx5*-KO BMSCs, showing both membrane and cytoplasmic localization, with quantification of membrane and cytoplasmic p-EGFR distribution. Yellow solid boxes indicate zoomed regions; white dashed lines outline cell boundaries (*n* = 3). Scale bar: 20 μm. **d** Immunofluorescence staining and magnified views of EGFR in WT and *Snx5*-KO BMSCs, with quantification of membrane-localized EGFR. Yellow solid boxes indicate zoomed regions; white dashed lines outline cell boundaries (*n* = 3). Scale bar: 20 μm. **e** Schematic diagram summarizing the subcellular distribution of EGFR and p-EGFR in WT and *Snx5*-KO BMSCs. **f** ALP and ARS staining of *Snx5*-KO BMSCs treated with EGF under static or tensile conditions. **g** Western blot analysis of p-EGFR, EGFR, and Osx protein levels in *Snx5*-KO BMSCs treated with EGF under static or tensile conditions. GSEA gene set enrichment analysis, FDR false discovery rate, ES enrichment score, NES normalized enrichment score, EGFR epidermal growth factor receptor, WT wild-type, KO knockout, ALP alkaline phosphatase, ARS alizarin red staining. Data are presented as mean ± SD. ns, not significant; *P < 0.05; **P < 0.01; ***P < 0.001; ****P < 0.000 1. Statistical analysis: **b**, **c** and **d** were assessed using a two-tailed Student’s *t* test

To explore the molecular mechanisms by which Snx5 regulates osteogenesis, gene set enrichment analysis (GSEA) was performed on the transcriptomic data of *Snx5*-KO BMSCs. The results revealed significant downregulation of EGFR signaling pathway in the *Snx5*-KO group (Fig. 4a). This was further validated by western blot analysis, which showed markedly reduced levels of total EGFR and its phosphorylated form, p-EGFR, in *Snx5*-KO BMSCs (Fig. 4b). Immunofluorescence staining demonstrated decreased membrane localization of EGFR, along with significantly diminished p-EGFR signals at the plasma membrane and in the cytoplasm (Fig. 4c, d). A schematic illustration summarizes the altered EGFR distribution in *Snx5*-KO BMSCs (Fig. 4e).

To confirm the role of EGFR signaling in Snx5-mediated osteogenesis, we treated *Snx5*-KO BMSCs with epidermal growth factor (EGF), an EGFR agonist. ALP and ARS staining revealed that EGF treatment suppressed the osteogenic capacity of *Snx5*-KO BMSCs under both static and mechanical stretch conditions (Fig. 4f). Correspondingly, western blot results showed that EGF restored p-EGFR expression while reducing levels of the osteogenic marker protein Osx (Fig. 4g).

**Fig. 5:**
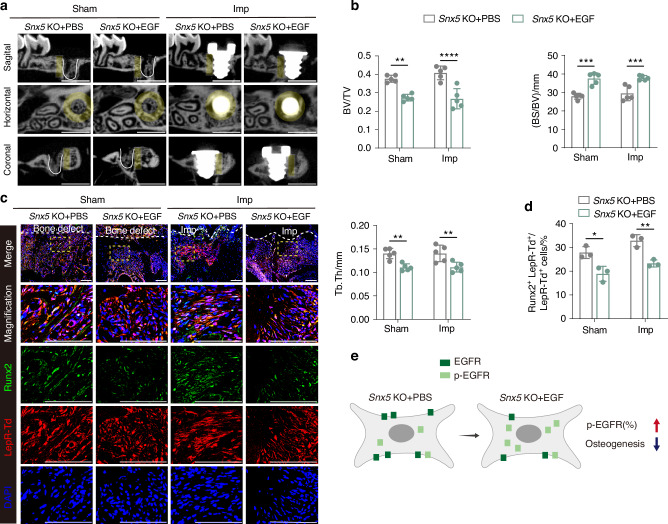
EGF-induced EGFR activation suppresses the enhanced osteogenic phenotype caused by Snx5 deficiency. **a** Representative sagittal, horizontal, and coronal micro-CT images of bone structure in *Snx5*-KO mice under Sham and Imp conditions following EGF treatment (*n* = 5). Yellow areas indicate ROIs, defined as annular zones extending approximately 75 μm to 175 μm outward from the implant surface; white solid lines delineate the defect boundaries in the Sham group. Scale bar: 1 mm. **b** Quantitative analysis of BV/TV, BS/BV, and Tb.Th (*n* = 5). **c** Immunofluorescence staining and magnified views of peri-defect or peri-implant regions in *Snx5*-KO mice after EGF treatment, showing Runx2 (green), LepR⁺ tdTomato⁺ (red), and DAPI (blue). Yellow dashed boxes indicate zoomed-in areas; white dashed lines delineate the defect or implant site boundaries (*n* = 3). Scale bars: 100 μm. **d** Quantification of Runx2⁺ LepR⁺ tdTomato⁺ cells as a percentage of total LepR⁺ tdTomato⁺ cells (*n* = 3). **e** Schematic illustration summarizing the regulatory effects of EGF on p-EGFR levels and osteogenic differentiation potential. Imp implant, EGFR epidermal growth factor receptor, KO knockout, ROI regions of interest, BV/TV bone volume fraction, BS/BV bone surface-to-volume ratio, Tb.Th trabecular thickness. Data are presented as mean ± SD. ns not significant. *P < 0.05; **P < 0.01; ***P < 0.001; ****P < 0.000 1. Statistical analysis: **b** and **d** were analyzed by two-way ANOVA followed by Tukey’s multiple comparisons test

In vivo, micro-CT analysis showed that EGF administration inhibited bone mass gain in *Snx5*-KO mice under both sham and implant placement conditions (Fig. 5a, b). Immunofluorescence analysis further demonstrated that EGF significantly increased the proportion of p-EGFR⁺ LepR⁺ tdTomato⁺ cells in peri-implant regions, while concurrently reducing the proportion of Runx2⁺ LepR⁺ tdTomato⁺ osteogenic cells (Figs. 5c, d and S6a, b). A schematic diagram illustrates the effects of EGF on EGFR signaling and osteogenic differentiation (Fig. 5e).

Collectively, these results suggest that Snx5 deficiency promotes osteogenesis by downregulating EGFR activity, whereas activation of EGFR signaling via EGF reverses this effect, highlighting EGFR as a critical downstream pathway mediating the regulatory role of Snx5 in osteogenesis.

### Snx5 regulates EGFR post-endocytic sorting, promoting its recycling and preventing lysosomal degradation

**Fig. 6:**
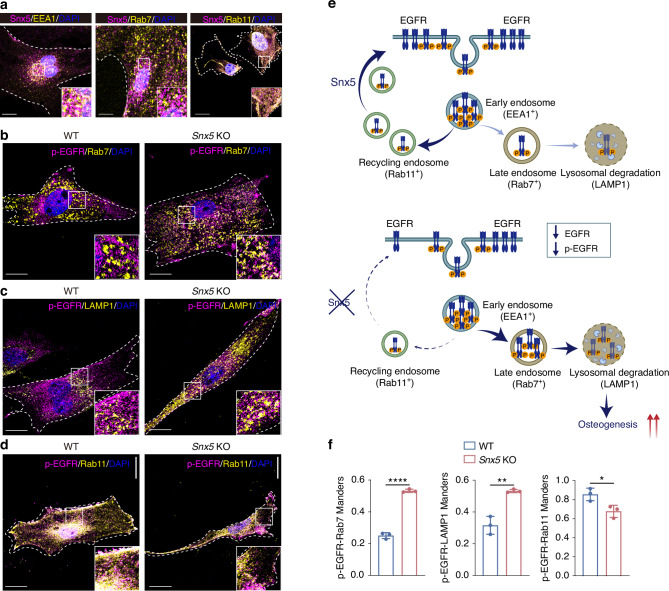
Snx5 regulates intracellular sorting of EGFR by promoting its recycling to the plasma membrane and preventing lysosomal degradation. **a** Immunofluorescence staining and magnified views showing co-localization of Snx5 (purple) with early endosomes (EEA1, yellow, left), late endosomes (Rab7, yellow, middle), and recycling endosomes (Rab11, yellow, right). White solid boxes indicate zoomed-in regions; white dashed lines delineate cell boundaries. Scale bar: 20 μm. **b** Immunofluorescence staining and magnified views of p-EGFR (purple) and Rab7 (yellow) in WT and *Snx5*-KO BMSCs. White solid boxes indicate zoomed-in regions; white dashed lines outline cell boundaries. Scale bar: 20 μm. **c** Immunofluorescence staining and magnified views of p-EGFR (purple) and LAMP1 (yellow) in WT and *Snx5*-KO BMSCs. White solid boxes indicate zoomed-in regions; white dashed lines outline cell boundaries. Scale bar: 20 μm. **d** Immunofluorescence staining and magnified views of p-EGFR (purple) and Rab11 (yellow) in WT and *Snx5*-KO BMSCs. White solid boxes indicate zoomed-in regions; white dashed lines outline cell boundaries. Scale bar: 20 μm. **e** Schematic diagram illustrating the proposed mechanism: in WT BMSCs, Snx5 facilitates the recycling of EGFR from early endosomes to the plasma membrane via Rab11⁺ recycling endosomes, maintaining EGFR signaling activity. In contrast, Snx5 deficiency redirects EGFR toward Rab7⁺ late endosomes and LAMP1⁺ lysosomes for degradation, resulting in reduced p-EGFR levels and enhanced osteogenesis. **f** Quantification of Manders’ overlap coefficients for p-EGFR with Rab7, LAMP1, and Rab11 (*n* = 3). WT wild-type, KO knockout. Data are presented as mean ± SD. ns not significant. *P < 0.05; **P < 0.01; ***P < 0.001; ****P < 0.000 1. Statistical analysis for **f** was performed using a two-tailed Student’s *t* test

To further elucidate the mechanism by which Snx5 regulates EGFR signaling, we reasoned that EGFR activity is tightly controlled by its intracellular trafficking, including endocytosis, recycling, and lysosomal degradation. Given that Snx5 is a member of the sorting nexin family involved in vesicle trafficking,^16^ we hypothesized that it may influence EGFR fate following endocytosis.^17^ We first assessed the subcellular localization of Snx5 in BMSCs via immunofluorescence co-staining with markers of three endosomal compartments: early endosomes (EEA1), late endosomes (Rab7), and recycling endosomes (Rab11) (Fig. 6a). The results showed prominent co-localization of Snx5 with EEA1 and Rab11, but minimal overlap with Rab7, indicating that Snx5 primarily localizes to early and recycling endosomes, potentially mediating the recycling of EGFR from early endosomes back to the plasma membrane.

Based on these localization findings, we examined how Snx5 deficiency affects intracellular EGFR sorting. Immunofluorescence staining revealed that in *Snx5*-KO cells, co-localization of p-EGFR with Rab7 and LAMP1 was significantly increased, whereas co-localization with Rab11 was markedly decreased (Fig. 6b–d). Quantitative analysis further supported these observations, suggesting that in the absence of Snx5, EGFR is preferentially directed toward Rab7⁺ late endosomes and subsequently degraded in LAMP1⁺ lysosomes, while recycling via Rab11⁺ endosomes back to the plasma membrane is impaired (Fig. 6f).

A mechanistic illustration summarizes the regulatory role of Snx5 in determining the intracellular fate of EGFR: in WT BMSCs, Snx5 facilitates EGFR recycling to the plasma membrane through Rab11⁺ endosomes, sustaining its signaling activity. In contrast, Snx5 deficiency disrupts this recycling route, redirecting EGFR toward lysosomal degradation and leading to reduced levels of p-EGFR (Fig. 6e).

### Inhibition of lysosomal degradation abrogates the enhanced osteogenic phenotype induced by Snx5 deficiency

**Fig. 7:**
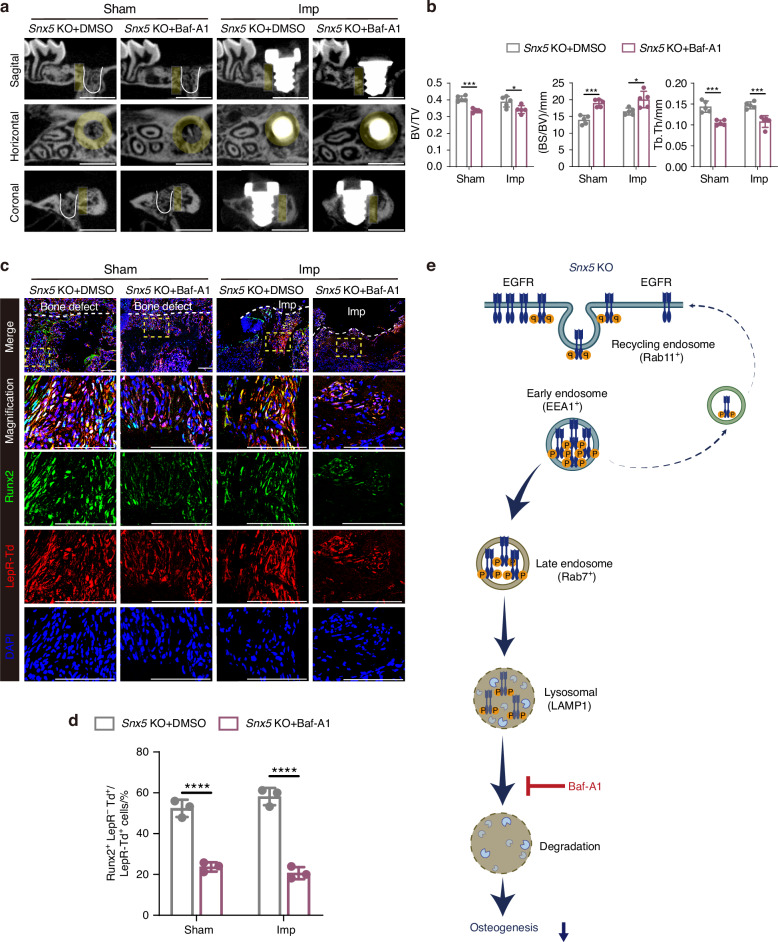
Inhibition of lysosomal degradation abolishes the enhanced osteogenic phenotype induced by Snx5 deficiency. **a** Micro-CT reconstructed images of bone architecture in *Snx5*-KO mice under Sham and Imp conditions after Baf-A1 treatment (*n* = 5). Yellow areas indicate ROIs, defined as annular zones extending approximately 75 μm to 175 μm outward from the implant surface; white solid lines delineate defect boundaries in the Sham group. Scale bar: 1 mm. **b** Quantitative analysis of BV/TV, BS/BV, and Tb.Th in *Snx5*-KO mice under Sham and Imp conditions after Baf-A1 treatment (*n* = 5). **c** Immunofluorescence staining and magnified views of peri-defect or peri-implant regions in *Snx5*-KO mice after Baf-A1 treatment, showing Runx2 (green), LepR⁺ tdTomato⁺ (red), and DAPI (blue). Yellow dashed boxes indicate zoomed-in areas; white dashed lines outline defect or implant site boundaries (*n* = 3). Scale bars: 100 μm. **d** Quantification of Runx2⁺ LepR⁺ tdTomato⁺ cells as a percentage of total LepR⁺ tdTomato⁺ cells (*n* = 3). **e** Schematic illustration of the proposed mechanism: in *Snx5*-KO cells, EGFR is primarily trafficked into Rab7⁺ late endosomes and degraded in LAMP1⁺ lysosomes. Treatment with the lysosomal inhibitor Baf-A1 blocks this pathway and attenuates the enhanced osteogenic phenotype. Baf-A1 Bafilomycin A1, Imp implant, ROI regions of interest. Data are presented as mean ± SD. ns not significant *P < 0.05; **P < 0.01; ***P < 0.001; ****P < 0.000 1. Statistical analysis for panels **b** and **d** was performed using two-way ANOVA followed by Tukey’s multiple comparisons test

Previous results suggest that in the absence of Snx5, EGFR is preferentially trafficked into LAMP1⁺ lysosomes via Rab7⁺ late endosomes for degradation. To determine whether lysosome-mediated degradation of EGFR directly contributes to the enhanced osteogenic phenotype observed in Snx5-deficient conditions, we treated cells with the lysosomal inhibitor Bafilomycin A1 (Baf-A1) (Fig. 7e). Immunofluorescence staining revealed a marked accumulation of p-EGFR within LAMP1⁺ lysosomes following Baf-A1 treatment, indicating effective blockade of EGFR degradation (Fig. S7a). Western blot analysis further showed that Baf-A1 treatment significantly upregulated p-EGFR expression in *Snx5*-KO BMSCs while suppressing the expression of Osx (Fig. S7b).

In vivo, micro-CT analysis demonstrated that Baf-A1 administration significantly inhibited the bone mass gain in *Snx5*-KO mice under both Sham and implant placement conditions, as evidenced by reduced BV/TV and Tb.Th values, and elevated BS/BV (Fig. 7a, b). Immunofluorescence staining showed that Baf-A1 treatment markedly increased the proportion of p-EGFR⁺ LepR⁺ tdTomato⁺ cells and significantly decreased the proportion of Runx2⁺ LepR⁺ tdTomato⁺ osteogenic cells (Figs. 7c, d and S7c, d).

These findings indicate that under Snx5-deficient conditions, EGFR is more prone to lysosomal degradation, and pharmacological inhibition of this pathway using Baf-A1 restores p-EGFR levels and counteracts the enhanced osteogenic phenotype. This highlights lysosomal degradation as a key mechanism through which Snx5 regulates EGFR signaling and bone formation.

## Discussion

In this study, we identified Snx5 as a novel regulator of the cellular response to mechanical stimulation. Integrated transcriptomic analyses of peri-implant LepR⁺ MSCs, peri-implant bone tissue, and BMSCs subjected to in vitro mechanical loading revealed consistent downregulation of Snx5 across all three sample types, suggesting a potential role for Snx5 in mechanotransduction. Functional assays indicated that Snx5 is essential for translating mechanical cues into osteogenic responses. Accordingly, in vivo Snx5 deficiency compromised implant-induced bone formation and blunted mechanosensitive activation of LepR⁺ cells. Mechanistically, we found that Snx5 is involved in mechanotransduction by modulating EGFR signaling axis. Specifically, Snx5 facilitates the recycling of endocytosed EGFR back to the plasma membrane, thereby limiting its lysosomal degradation and preserving downstream signaling activity. Snx5 deficiency leads to sustained EGFR degradation, resulting in elevated basal osteogenesis but impaired mechanoresponsiveness of BMSCs. This highlights a critical role for Snx5 in calibrating osteogenic responses to mechanical stimuli.

Traditionally, the maintenance of jawbone mass has been attributed to cyclic mechanical stimulation generated by occlusal forces. However, clinical observations combined with our experimental results reveal that even in the absence of functional loading, implant placement alone can effectively preserve local alveolar bone volume.^5,6^ This suggests that the biological effects of mechanical stimuli extend beyond direct loading and may involve modulation of bone homeostasis through alterations in endogenous tensile strain. In this study, we focused our analysis on regions of concentrated peri-implant stress while deliberately excluding implant-contact zones and CT regions potentially affected by partial volume effects. This strategy minimized the interference from material properties and imaging artifacts.^18–20^ Importantly, we found that Snx5 deficiency significantly affected the expression of early osteogenic markers Runx2 and Osx in peri-implant bone remodeling zones. These areas correspond well with previously reported sites of early remodeling activity in rodent models of implantation.^21^ This finding suggests that Snx5 may participate in a peri-implant specific remodeling mechanism by activating localized osteogenic programs. Notably, this effect appears independent of the material properties of the implant itself, highlighting a critical role for Snx5 in sensing the peri-implant microenvironment and orchestrating bone remodeling.

In both in vivo and in vitro models, mechanical stimulation failed to further enhance osteogenic responses in *Snx5*-KO cells or mice. This finding suggests that loss of Snx5 impairs the capacity of BMSCs to sense or transduce mechanical cues, highlighting an essential role for Snx5 in mechanotransduction rather than in basal osteogenesis. Notably, this pattern closely resembles findings from a study by Wang L et al.,^22^ in which conditional deletion of *Piezo1* in Prrx1-lineage cells resulted in a “mechanical desensitization” phenotype—wherein the bone mass of trabecular regions remained largely unchanged before and after unloading stimuli. Together, these results suggest a convergent mechanism wherein the inactivation of critical mechanosensors or transducers—such as Snx5 in BMSCs or Piezo1 in osteoblast-lineage cells—disrupts the ability of skeletal tissues to adapt to changes in the mechanical environmental.

Furthermore, we identified a pivotal role of Snx5 in regulating EGFR signaling. EGFR is a transmembrane tyrosine kinase receptor that undergoes dimerization upon ligand binding to its extracellular domain, thereby activating its intracellular kinase domain and initiating downstream signaling cascades.^23^ Our data showed that knockout of Snx5 significantly reduced the levels of EGFR and p-EGFR, suggesting that Snx5 may be involved in post-endocytic sorting of EGFR. This sorting process is crucial for EGFR signaling maintenance,^24^ as activated EGFR can either be recycled back to the plasma membrane to sustain signaling or targeted for lysosomal degradation. The balance between these two routes is essential for signal attenuation and cellular homeostasis. Our findings demonstrate that Snx5 facilitates the trafficking of p-EGFR into recycling endosomes, enabling its return to the plasma membrane and thereby maintaining signaling integrity and activity. In contrast, loss of Snx5 promotes the preferential trafficking of EGFR toward late endosomes and lysosomal degradation, disrupting the negative regulation of EGFR signaling. This mechanism was validated in both in vivo implant models and in vitro mechanical loading systems.

Although we observed a downward trend in the expression of *Piezo2* and *Yap1* in *Snx5-*KO cells, the differences did not reach statistical significance. This suggests that the mechanosensory function of Snx5 may not rely on directly regulating the expression levels of classical mechanosensitive molecules such as Piezo2 or Yap1. However, this finding does not exclude the possibility that Snx5 may exert its regulatory role through direct or indirect interaction with Piezo2 or Yap1. For example, in a yeast two‑hybrid screening‑based preprint study, Snx5 was identified as a potential interaction candidate for piezo channels,^25^ suggesting that Snx5 might have a direct functional association with members of the piezo family. In addition, previous studies have demonstrated extensive cross‑regulation between EGFR and Yap1 signaling. EGFR can modulate Yap1 activity through downstream PI3K–AKT and MAPK pathways, while Yap1,^26^ in turn, can regulate transcription factors such as TEAD and AP-1 to enhance EGFR gene expression and thereby increase cellular sensitivity to EGFR signaling.^27^ Combined with our current findings that Snx5 modulates EGFR signaling activity to regulate cellular responses to mechanical stimuli, we hypothesize that Snx5 may influence Yap1 activity indirectly through EGFR‑mediated signaling. This potential regulatory mechanism warrants further investigation in future studies.

Indeed, the SNX protein family plays a critical role in intracellular membrane trafficking and receptor transport.^28^ Various members of this family have been widely reported to participate in protein sorting and recycling between organelles. For example, Snx10,^29^ a known family member, has been shown to regulate the endosomal recycling and trafficking of the human epidermal growth factor receptor 2 (HER2). Loss of Snx10 results in HER2 accumulation within lysosomes, thereby reducing its surface expression and impairing the efficacy of anti-HER2 therapies. In our study, the Snx5-mediated sorting and trafficking of EGFR observed in peri-implant BMSCs closely resembles the regulatory mechanism employed by other SNX family proteins.

Notably, in addition to Snx5, EGFR itself has been implicated in mechanotransduction. A study by Li et al.^30^ reported that upon sensing mechanical stress, Piezo1 can trigger EGFR endocytosis via calcium influx, thereby activating the ERK signaling cascade to mediate cell cycle re-entry and proliferation. Moreover, ligand-dependent activation of EGFR has been shown to lower the mechanical threshold required for integrin activation, thereby enhancing cell spreading, traction force generation, and focal adhesion maturation.^31^ These findings suggest that EGFR is not only a pivotal regulator of cell proliferation but also a potential integrator of mechanical signals. Combined with our current findings, these observations further support the existence of a Snx5–EGFR axis that connects mechanical input to BMSCs fate decisions, particularly within the mechanically dynamic environment induced by dental implant placement in the jawbone.

This study is the first to identify Snx5, a member of the sorting nexin family, as a novel key regulator that mediates the response of peri-implant jawbone BMSCs to mechanical stimulation and governs their osteogenic fate decisions, thereby extending the known functional repertoire of the SNX family. This finding provides a new perspective for exploring the role of mechanical environments in bone regeneration. By modulating Snx5 activity, it may be possible to precisely regulate the bone mechanical microenvironment, thereby promoting bone healing and regeneration and offering new therapeutic avenues in clinical practice.

## Materials and methods

### Mice

Wild-type mice with a C57BL/6 background were procured from Leigen Biotech Company (Shanghai, China). The B6.129(Cg)-LepRtm2(cre)Rck/J (LepR-Cre) (Strain #:008320) and B6;129S6-Gt(ROSA)26Sortm9(CAG-tdTomato)Hze/J (Rosa26-tdTomato) (Strain #:007905) mice with C57BL/6 background were purchased from the Jackson Laboratory. C57BL/6JCya-*Snx5*em1/Cya (*Snx5*-KO) mouse (S-KO-16307) with C57BL/6J background was obtained from Cyagen Biotech Company (Suzhou, China). In the knockout model used in this study, exons 4 to 13 of the *Snx5* gene—accounting for approximately 77.97% of the coding sequence and encompassing regions critical for functional domains of Snx5—were deleted using CRISPR/Cas9 technology, resulting in a functional knockout (Fig. S3). *Snx5*-KO mice were crossed with LepR-Cre and Rosa26-tdTomato mice to generate LepR-tdTomato; *Snx5*-KO mice. All mice were housed at the Laboratory Animal Center of Tongji University with constant temperature ((22±2) °C) and humidity ((55±10) %) in a 12-h light/dark cycle. All animal studies were approved by the Institutional Animal Care and Use Committee of Tongji University (approval number: [2022]-DW-10).

### Cone-beam computed tomography (CBCT) data selection and analysis

To ensure the representativeness of the data and exclude potential confounding factors, we established the following inclusion and exclusion criteria for the participants:

**Inclusion criteria:**

1. Healthy adults aged 25–45 years;
2. No gender restrictions and no history of systemic diseases;
3. Extraction of a single mandibular first molar with an intact socket wall;
4. No history of or ongoing orthodontic treatment;
5. No metabolic bone diseases;
6. No smoking or alcohol abuse.

**Exclusion criteria:**

1. Any systemic diseases affecting bone metabolism;
2. Presence of acute or chronic infection foci in the oral cavity;
3. Use of medications affecting bone metabolism;
4. Individuals with CBCT image quality insufficient for subsequent analysis.

Twenty patients were selected for each group, and CBCT data were collected. In this study, the CBCT scanner (NewTom) was configured with a voltage of 110 kV and an exposure time of 3.6 s. All participants provided informed consent for the use of their CBCT data. Bone morphometric parameters were analyzed using Dragonfly software (Object Research Systems Inc.), and alveolar bone quality was assessed by measuring alveolar bone height.

### RNA sequencing and analysis

Total RNA was extracted by treating LepR^+^ MSCs, derived from both WT and *Snx5*-KO mice, with RNAiso Plus. Each sample was collected from the maxilla of three mice. The RNA purity was assessed using a NanoVue (GE, USA) and the A260/A280 ratio of each sample was maintained at >1.90. Agilent 2200 TapeStation (Agilent Technologies) was used for the evaluation of RNA integrity with the RNA integrity number (RIN) > 7.0. The library preparation was carried out according to the instructions provided with the RNA-Seq Library Preparation Kit (NR606, Vazyme, China). Subsequently, the purified products were evaluated using the Agilent 2200 TapeStation and Qubit®2.0 (Life Technologies, USA), followed by sequencing using a NovaSeq X Plus platform (PE150).

In analysis, after removing the raw reads of low quality, HISAT2 was used to align the remaining clean reads to the mouse genome database (mm10) or human genome database (hg38) in accordance with the species. For the selection of target genes in Fig. 1e, we chose three datasets: the first group (GSM7109973 vs GSM7498409), the second group (PRJNA924055 vs GSE154748), and the third group (GSE112122). Following the steps mentioned above, we obtained the count files for each dataset and merged them into expression matrices for each group. Differential gene expression was calculated using the DESeq2 package (v1.42.1).^32^ Differentially expressed genes for each group were selected based on the following criteria: 1. average expression level in the top 20%; 2. Log_2_FC > 0.5 or Log_2_FC < –0.5; 3. padj <0.001. Differential gene selection for Fig. 2 was performed using a similar approach, but with selection criteria: Log_2_FC > 1 or Log_2_FC < –1,–log10(FDR)  > 2. PCA was performed using the FactoMineR package (v1.42).^33^ For Fig. 2a, all genes with TPM > 10 were used for PCA; for Fig. 2c–e, genes shown in the bar charts were used for PCA. The enrichment of functional gene pathways and biological processes of gene ontology was assessed by GSEA using the KEGG pathway database (c2.cp.kegg.v6.0.symbols.gmt) and GO-BP (c5.bp.v6.0.symbols.gmt).

### Establishment of the mouse implant model

Following anesthesia induced by ketamine (80 mg/kg) and xylazine (16 mg/kg), the bilateral maxillary first molars were extracted. The sites were drilled to a diameter of 0.6 mm at their original locations. A single side was randomly chosen for the insertion of a prefabricated titanium nail (0.65 mm in diameter, 1 mm in length; Weigao, China), and a torque meter (45ATG; Tohnichi, Japan) was utilized to maintain the torque at 5 gf·cm.^34^ On the contralateral side, which was designated the Sham group, expansion holes without the insertion of titanium nails were created. Osseointegration was largely achieved approximately 14 days post-implantation in mice, with samples collected on postoperative day 14 for this study.^35^ To activate EGFR, recombinant EGF protein (HY-P7067, MCE) was locally administered at a dose of 0.2 mg/kg on alternate days.^36^ In vivo, Bafilomycin A1 (Baf-A1) (HY-100558, MCE) was dissolved in DMSO, diluted with saline, and administered intraperitoneally at a dose of 1 mg/kg.^37,38^

### Micro-CT

After euthanizing the mice, the jawbones were extracted and subjected to micro-CT scanning (CT50; SCANCO Medical) at a resolution of 14.8 μm. Two-dimensional (2D) images of maxillary scan slices, reconstructed three-dimensional (3D) microscopic images, and calculated structural indices were obtained using ancillary analysis software. The region of interest (ROI) for maxillary tissue was defined as an annular region surrounding the implant, extending approximately 75 μm to 175 μm outward from the implant surface to capture peri-implant trabecular bone changes. The parameters calculated in this study included BV/TV, BS/BV, and Tb.Th within the ROI.

### Immunofluorescence staining of tissue sections

The mice were anesthetized, and cardiac perfusion was performed. The maxilla was then excised and fixed in 4 % paraformaldehyde (PFA) at 4 °C for 24 h, followed by decalcification in a 10 % ethylenediaminetetraacetic acid solution for 4 weeks. Upon completion of decalcification, the implants were meticulously removed from the maxilla.^32^ The samples were embedded in paraffin and sectioned at 4 μm thickness. For IF staining of the sections, microwave-assisted antigen retrieval was conducted using a citrate antigen retrieval solution (P0081; Beyotime Biotechnology). Sections were blocked using powerblock, and the primary antibodies were incubated at 4 °C overnight following a 10-min block period, with negative controls prepared by replacing the primary antibodies with PBS. After the removal of the primary antibodies, the sections were incubated with secondary antibodies at room temperature in the dark for 1 h. The primary antibodies and secondary antibodies used in this experiment are listed in Table S2. Nuclei were stained with DAPI (1:1 000; C0065; Beyotime) for 10 min, and the coverslips were sealed with an antifade mounting medium. Observation and imaging were performed using a laser confocal microscope (Nikon, Japan). Data analysis was conducted with ImageJ software (National Institutes of Health, USA).

### Cell immunofluorescence staining

Cell coverslips of corresponding diameters were placed in 24-well cell culture plates, and LepR^+^ MSCs were seeded onto the coverslips at a density of 2000 cells per well for culture. Cells were fixed with 4 % PFA for 30 min, followed by permeabilization with 0.2 % Triton X-100 for 10 min. Cells were blocked using powerblock, and after a 10-min blocking period, the primary antibodies were incubated overnight at 4 °C. Subsequent procedures were performed as described for tissue immunofluorescence staining. The primary antibodies and secondary antibodies used in this experiment are listed in Table S2.

### Extraction and culture of BMSCs

BMSCs were extracted from 3-week-old LepR-Cre; tdTomato mice as described previously.^39^ Following euthanasia, the soft tissues and teeth were meticulously excised from the jawbone surfaces. The jawbones were carefully sectioned into small fragments and subjected to enzymatic digestion with 1.5 mL of 2 mg/mL type I collagenase (17100017; Gibco) at 37 °C with continuous agitation at 300 r/min for 2 h. After centrifugation at 1 200 r/min for 5 min, the cell pellet was resuspended in α-MEM (SH30265.01; HyClone) supplemented with 10 % fetal bovine serum (FBS; 04-001-1ACS; BI) and 1 % penicillin–streptomycin (SV30010; HyClone) and then seeded in 10 cm diameter cell culture dishes. The cells were cultured at 37 °C and 5 % CO_2_, and the medium was changed every 3 days until the cells reached 80 % confluence for passaging. Third-passage BMSCs were used in this study.

### Flow cytometry

BMSCs isolated from LepR-Cre; tdTomato mice were cultured to passage 3, after which the cells were digested and centrifuged at 1 200 r/min for 5 min to harvest the cells. Subsequently, 400 µL of Cell Staining Buffer (Biolegend, USA) was added to the cells, which were then analyzed using a BD LSRFortessa Flow Cytometer (BD Biosciences, USA). FlowJo 10.8.1 software (Ashland, OR, USA) was used to analyze the data.

### Osteogenic differentiation of BMSCs

BMSCs were seeded at a density of 5 × 10^5^ or 2.5 × 10^5^ cells per well in 6-well or 12-well plates, and osteogenic induction medium was prepared by adding 10 % FBS, 1 % penicillin–streptomycin, 10 mmol/L β-glycerophosphate sodium (G9422; Sigma), 100 nmol/L dexamethasone (D4902; Sigma), and 50 μg/mL ascorbic acid (A8960; Sigma) to the α-MEM. After cell attachment, the medium was changed to an osteogenic induction medium. In the Baf-A1 treatment group, 1 nmol/L Baf-A1 (HY-100558, MCE) was incorporated into the osteogenic induction medium, while an equivalent volume of DMSO (D103274, Aladin) was administered to the control cells. The EGF treatment group received supplementation with 0.01 nmol/L EGF recombinant protein (HY-P7067, MCE) solubilized in non-enzymatic water within the osteogenic induction medium, and an equal volume of PBS buffer was added to the control cells. Alkaline phosphatase (ALP; C3206; Beyotime Biotechnology) staining was performed after 7 days of osteogenic induction, total cellular protein was extracted after 14 days, and both Alizarin Red S (ARS; A5533; Sigma) staining and von Kossa (G3282; Solarbio) staining were conducted after 28 days to evaluate late-stage matrix mineralization. Staining procedures were carried out according to the instructions.

### Application of tension

The cells were subjected to mechanical tension using the stretch module of Flexcell FX-5000T system. BMSCs were seeded at a density of 5 × 10^5^ cells/well on type I collagen-coated BioFlex plates (BF-3001C; Flexcell) and cultured with osteogenic induction medium under mechanical tension (10 %, 0.5 Hz, 24 h) every other day for 7 days. Control cells were seeded on the same type of tension plates and cultured under identical conditions but without the application of mechanical stimulation.

### Western blot

Cells were lysed in radioimmunoprecipitation assay (RIPA) buffer containing protease inhibitors (P1046; Beyotime Biotechnology) for 30 min to collect total protein. Each protein sample was separated by sodium dodecyl sulfate-polyacrylamide gel electrophoresis (SDS–PAGE) and transferred to a polyvinylidene difluoride (PVDF) membrane. The membrane was blocked and incubated with primary antibodies overnight at 4 °C, followed by incubation with HRP-conjugated secondary antibodies for 1 h. The primary antibodies and secondary antibodies used in this experiment are listed in Table S2. Finally, images were obtained using a chemiluminescence system (Cytiva, USA), and Fiji (ImageJ) software was used for relative quantitative analysis.

### Statistics

The data are reported as the mean ± SD, with each experiment comprising a minimum of three biological replicates for statistical analysis. In vivo, samples were randomly assigned, and representative images were selected for presentation. Statistical analyses were conducted via Prism software (GraphPad Software, USA). For data that were normally distributed with homogeneous variance, unpaired Student’s *t* tests were employed for comparisons between two groups, and two-way analysis of variance followed by post hoc tests were utilized for comparisons among multiple groups. For data not normally distributed or exhibiting heterogeneous variances, the Kruskal–Wallis test followed by multiple comparisons was used for more than three groups, and the Mann–Whitney U test was applied for two-group comparisons.

## Supplementary information

Supplementary information

## Data Availability

The raw/processed data required to reproduce these findings can be shared upon reasonable request. RNA-seq data have been deposited in the Gene Expression Omnibus (GEO) database (GSE280673). The data that support the findings of this study are available on request from the corresponding author.
