# Supplementary material for: Implantation awakens peri-implant osteogenic potential via Snx5-EGFR axis-mediated mechanical transduction
Source: Int J Oral Sci. 2026 Feb 20;18:18. doi: 10.1038/s41368-025-00423-2 (PMC12920663; doi:10.1038/s41368-025-00423-2)
Supplement: Supplementary file 1 — Supplementary information [file 41368_2025_423_MOESM1_ESM.docx]

Supplementary Figures


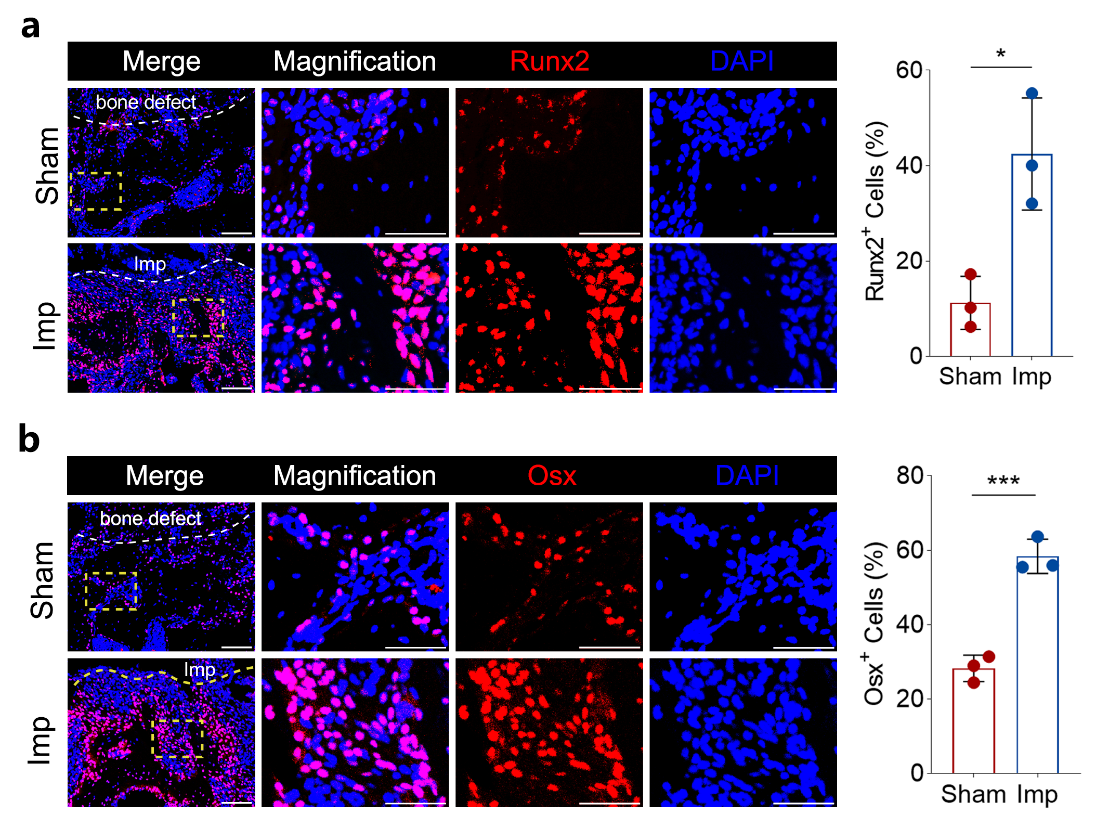


**Figure S1. Dental implant placement promotes osteogenesis.**

**a** Immunofluorescence staining and quantification of Runx2⁺ cells in peri-defect or peri-implant tissues from Sham and Imp groups. Overview and magnified views are shown on the left, with yellow dashed boxes indicating zoomed regions and white dashed lines delineating the defect or implant site boundaries (n = 3). Scale bars: 100 μm.

**b** Immunofluorescence staining and quantification of Osx⁺ cells in peri-defect or peri-implant tissues from Sham and Imp groups. Overview and magnified views are shown on the left, with yellow dashed boxes indicating zoomed regions and white dashed lines delineating the defect or implant site boundaries (n = 3). Scale bars: 100 μm.

Imp, Implant;

Data are presented as mean ± SD. *p < 0.05; ***p < 0.001. Statistical analysis: two-tailed Student’s t-test for panels a and b.


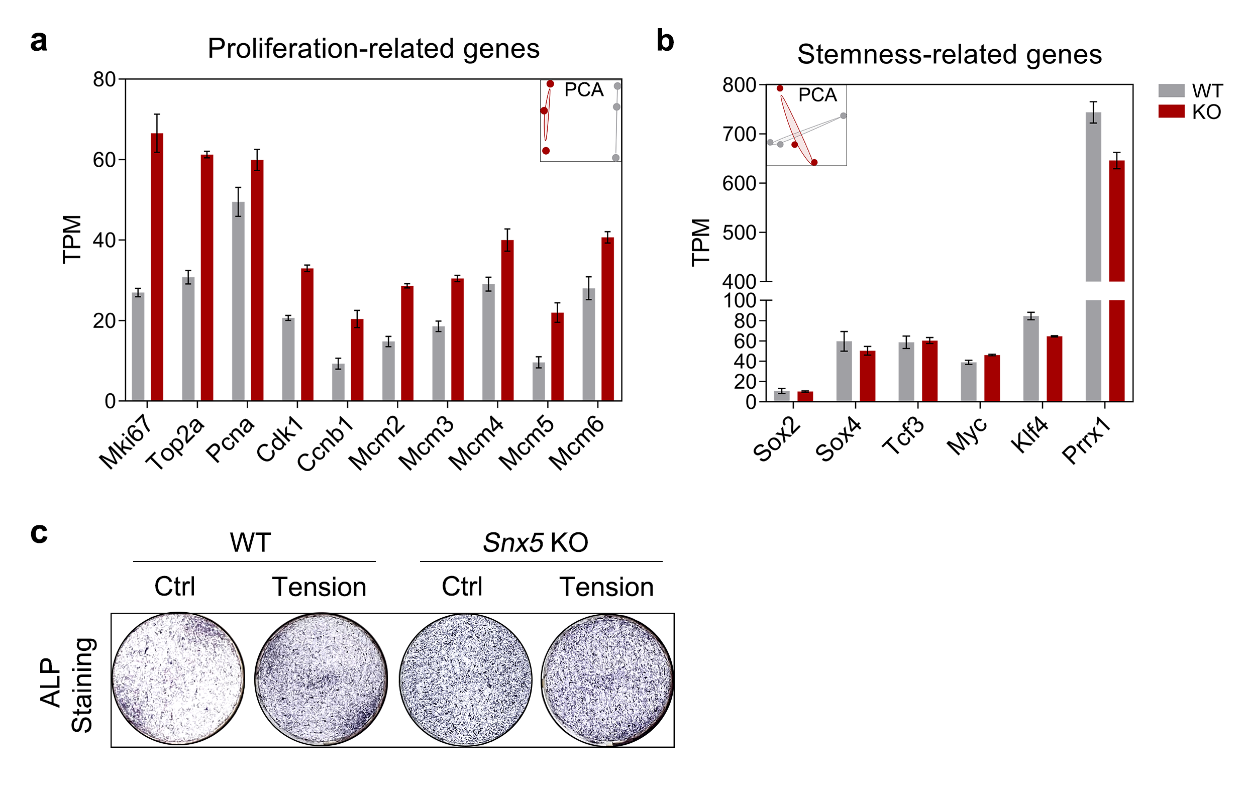


**Figure S2. Snx5 deficiency reshapes the transcriptomic profile of BMSCs, enhances basal osteogenesis, but impairs osteogenic responses to mechanical stimulation.**

**a, b** TPM values for genes associated with proliferation, and stemness between WT and *Snx5*-KO BMSCs.

**c** ALP staining of WT and *Snx5*-KO BMSCs under static or tensile strain conditions.

WT, wild-type; KO, knockout; TPM, Transcripts per million; ALP, Alkaline phosphatase


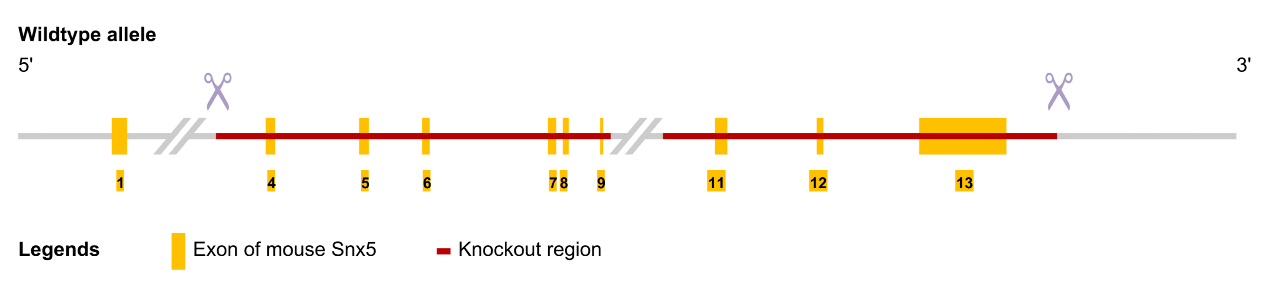


**Figure S3. Schematic diagram of *Snx5* knockout strategy in mice.**


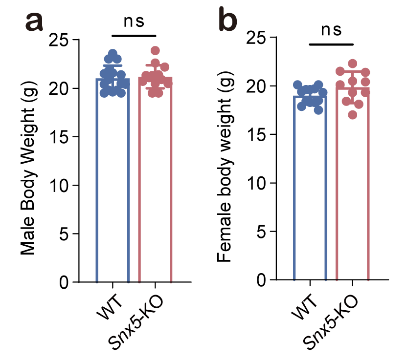


**Figure S4. Body weight of *Snx5*-KO and WT mice at 8 weeks of age.**

**a** Body weight comparison between male WT and *Snx5*-KO mice (n = 13).

**b** Body weight comparison between female WT and *Snx5*-KO mice (n = 11).

Data are presented as mean ± SD. ns, not significant. Statistical analysis: two-tailed Student’s t-test for panels a and b.


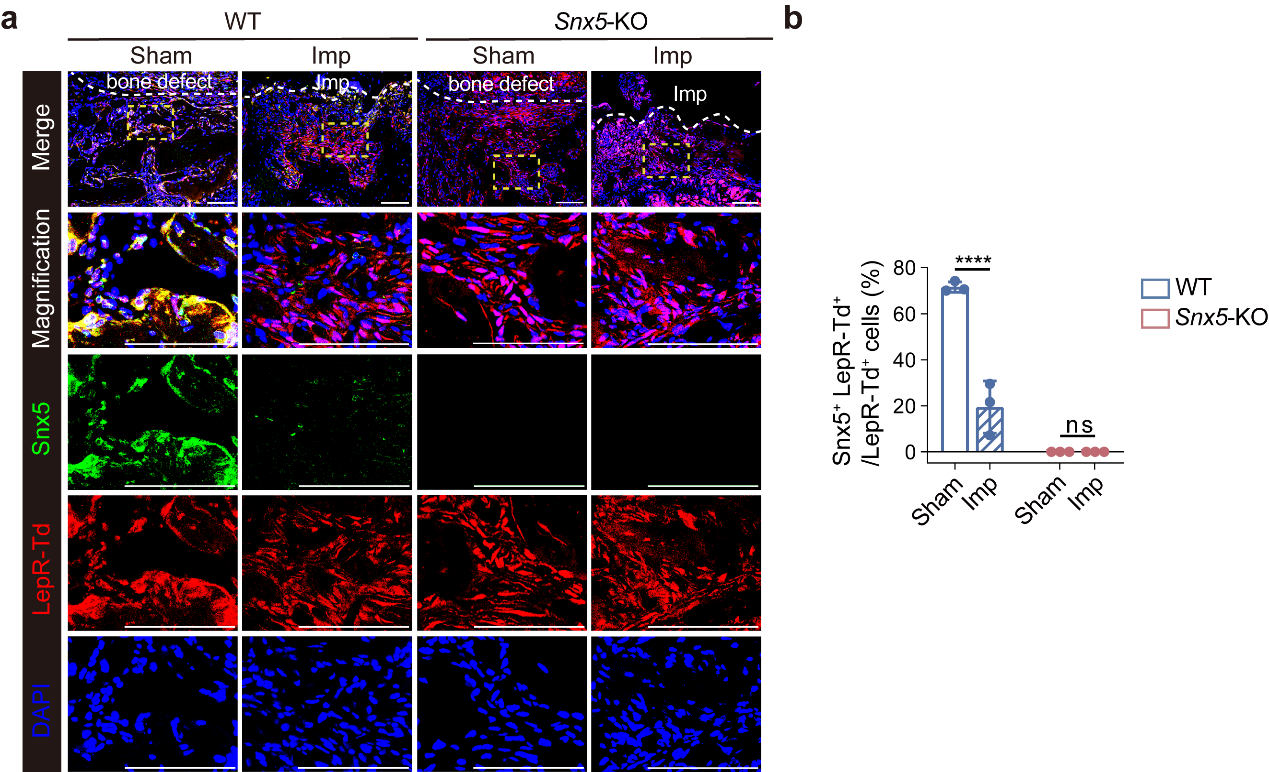


**Figure S5. Validation of *Snx5* KO in LepR⁺ cells.**

**a** Immunofluorescence staining and magnified views of peri-defect or peri-implant regions from Sham and Imp groups, showing Snx5 (green), LepR⁺tdTomato⁺ (red), and DAPI (blue). Yellow dashed boxes indicate zoomed-in areas; white dashed lines outline the boundaries of the defect or implant sites (n = 3). Scale bars: 100 μm.

WT, wild-type; KO, knockout; Imp, Implant; ROIs, regions of interest;

Data are presented as mean ± SD. ns, not significant; ****p < 0.0001. Statistical analysis for panels b was performed using two-way ANOVA followed by Tukey’s multiple comparisons test.


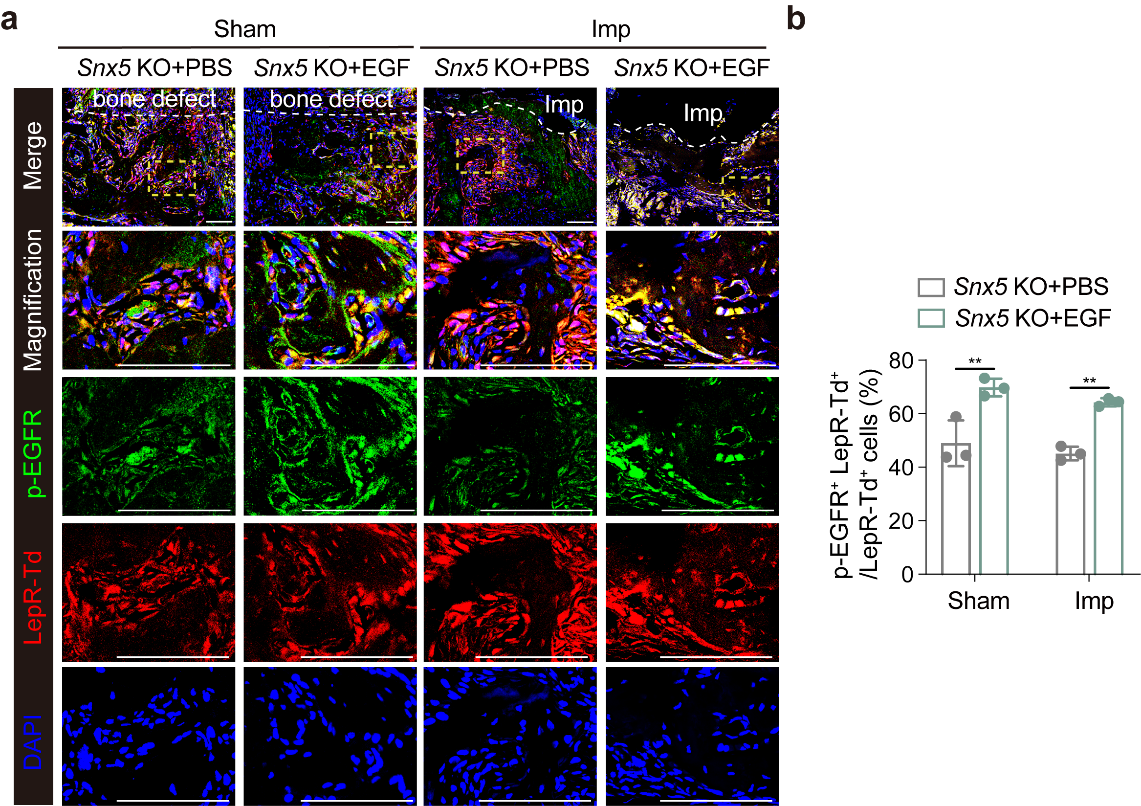


**Figure S6. EGF treatment activates EGFR signaling in LepR⁺ cells.**

**a** Immunofluorescence staining and magnified views of peri-defect or peri-implant regions in *Snx5*-KO mice after EGF treatment, showing p-EGFR (green), LepR⁺tdTomato⁺ (red), and DAPI (blue). Yellow dashed boxes indicate zoomed-in areas; white dashed lines delineate the defect or implant site boundaries (n = 3). Scale bars: 100 μm.

EGFR, epidermal growth factor receptor; KO, knockout; ROIs, regions of interest;

Data are presented as mean ± SD. **p < 0.01. Statistical analysis: panels b was analyzed by two-way ANOVA followed by Tukey’s multiple comparisons test.


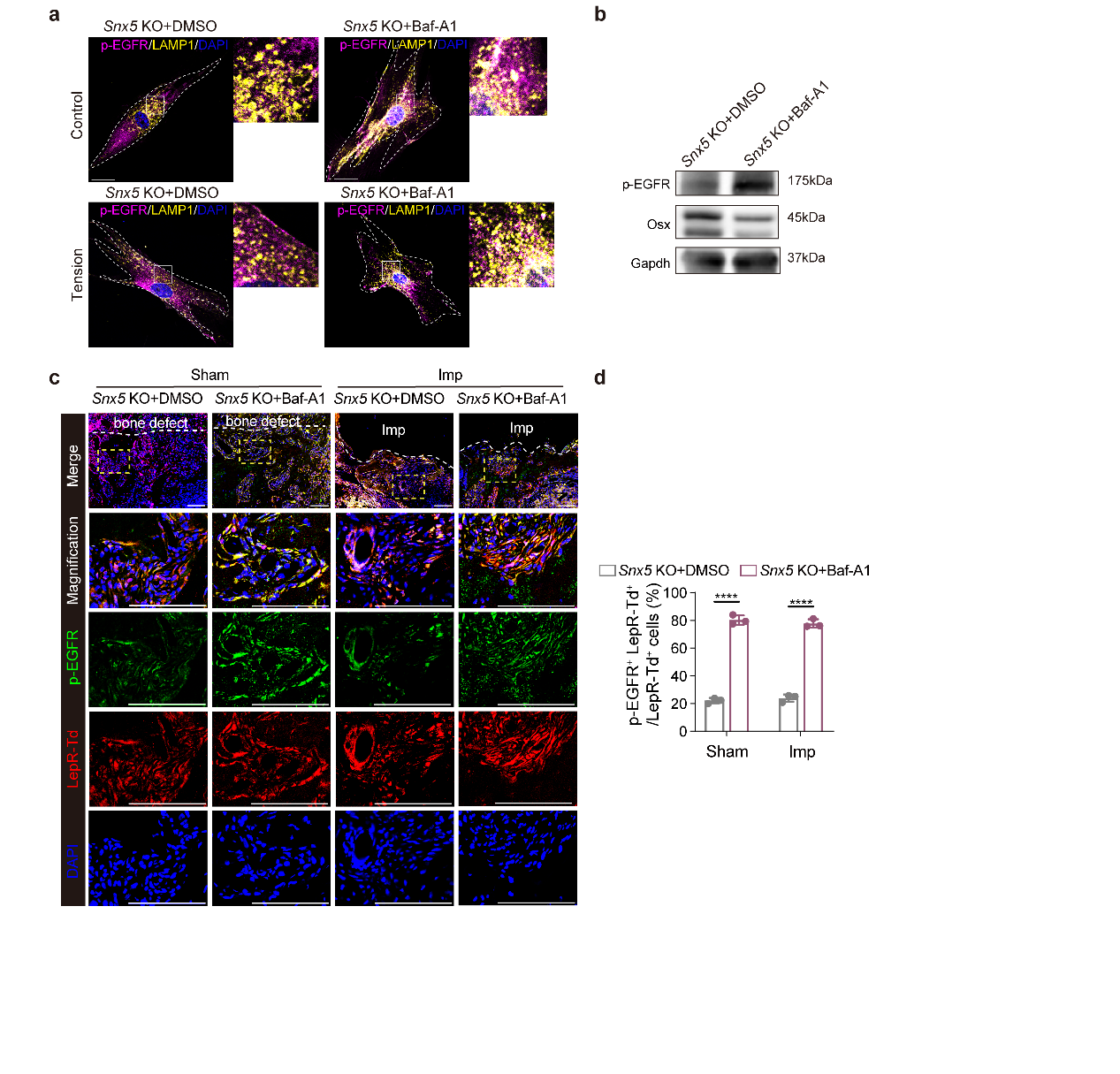


**Figure S7. Baf-A1 treatment activates EGFR signaling in LepR⁺ cells.**

**a** Immunofluorescence staining and magnified views of p-EGFR (purple) and LAMP1 (yellow) in *Snx5*-KO BMSCs under static and tensile conditions following Baf-A1 treatment. White solid boxes indicate zoomed regions; white dashed lines outline cell boundaries. Scale bar: 20 μm.

**b** Western blot analysis showing the effects of Baf-A1 treatment on p-EGFR and Osx protein levels in *Snx5*-KO BMSCs.

**c** Immunofluorescence staining and magnified views of peri-defect or peri-implant regions in *Snx5*-KO mice after Baf-A1 treatment, showing p-EGFR (green), LepR⁺ tdTomato⁺ (red), and DAPI (blue). Yellow dashed boxes indicate zoomed-in areas; white dashed lines outline defect or implant site boundaries (n = 3). Scale bars: 100 μm.

Baf-A1, Bafilomycin A1; Imp, Implant; ROIs, regions of interest;

Data are presented as mean ± SD. ****p < 0.0001. Statistical analysis for panels d was performed using two-way ANOVA followed by Tukey’s multiple comparisons test.

Supplementary Tables

**Table S1. Sequences of mouse primers for genotyping.**

| Primers for genotyping | |
| --- | --- |
| *Snx5^-/-^-F* | GCAGAATTAAGAATCTGCCCAGTAGT |
| *Snx5^-/-^-R* | CAGTTTTGAACTAGGCTCTGCCTG |
| *LepR-Cre-F* | GCTGGAAGATGGCGATTAGC |
| *LepR-Cre-R* | TCTTCTTTCCAGAGTTCAGATGT |
| *Td-tomato-F* | GGCATTAAAGCAGCGTATCC |
| *Td-tomato-R* | CTGTTCCTGTACGGCATGG |

**Table S2. Antibodies.**

|  | **Antibodies** | **Sources** | **Cat. No.** | **Dilutions** | **Applications** |
| --- | --- | --- | --- | --- | --- |
| 1 | Anti-Runx2 | Abcam | ab192256 | 1:200 | IF |
|  |  |  |  | 1:1000 | WB |
| 2 | Anti-Osx | Abcam | ab209484 | 1:200 | IF |
|  |  |  |  | 1:1000 | WB |
| 3 | Anti-Alpl | BOSTER | A01008-1 | 1:1000 | WB |
| 4 | Anti-Snx5 | Abcam | ab180520 | 1:100 | IF |
|  |  |  |  | 1:1000 | WB |
| 5 | Anti-EGFR | Abcam | ab52894 | 1:1000 | WB |
| 6 | Anti-p-EGFR | Abcam | ab40815 | 1:100 | IF |
|  |  |  |  | 1:1000 | WB |
| 7 | Anti-Rab7 | Santa Cruz | sc-376362 | 1:100 | IF |
| 8 | Anti-LAMP1 | eBioscience | 14-8071-82 | 1:100 | IF |
| 9 | Anti-Rab11 | Proteintech | 67902-1-Ig | 1:100 | IF |
| 10 | Anti-EEA1 | R&D | AF8047-SP | 1:100 | IF |
| 11 | Anti-LepR | R&D | AF497-SP | 1:100 | IF |
| 12 | Anti-RFP | Chromotek | 5f8 | 1:200 | IF |
| 13 | Alexa Fluor 488-conjugated donkey anti-rabbit secondary antibody | Thermo Fisher Scientific | A-21206 | 1:1000 | IF |
| 14 | Alexa Fluor 594-conjugated donkey anti-rabbit secondary antibody | Thermo Fisher Scientific | A-21207 | 1:1000 | IF |
| 15 | Alexa Fluor 488-conjugated donkey anti-Goat secondary antibody | Thermo Fisher Scientific | A-11055 | 1:1000 | IF |
| 16 | HRP-conjugated goat anti-rabbit secondary antibodies | Beyotime Biotechnology | 0208 | 1:1000 | WB |
| 17 | HRP-conjugated goat anti-mouse secondary antibodies | Beyotime Biotechnology | 0216 | 1:1000 | WB |
